# Analysis of gene expression profiles and protein-protein interaction networks in multiple tissues of systemic sclerosis

**DOI:** 10.1186/s12920-019-0632-2

**Published:** 2019-12-27

**Authors:** Elham Karimizadeh, Ali Sharifi-Zarchi, Hassan Nikaein, Seyedehsaba Salehi, Bahar Salamatian, Naser Elmi, Farhad Gharibdoost, Mahdi Mahmoudi

**Affiliations:** 1 Rheumatology Research Center, Tehran University of Medical Sciences Shariati Hospital, Kargar Ave, P.O. BOX 1411713137, Tehran, Iran; 2 Department of Computer Engineering, Sharif University of Technology, Azadi Ave, P.O. BOX 11365-11155, Tehran, Iran; 3 Department of Mathematical Sciences, Sharif University of Technology, Tehran, Iran; 4 Inflammation Research Center, Tehran University of Medical Sciences, Tehran, Iran

**Keywords:** Systemic sclerosis, Functional analysis, Common pathway, Integrative gene expression analysis

## Abstract

**Background:**

Systemic sclerosis (SSc), a multi-organ disorder, is characterized by vascular abnormalities, dysregulation of the immune system, and fibrosis. The mechanisms underlying tissue pathology in SSc have not been entirely understood. This study intended to investigate the common and tissue-specific pathways involved in different tissues of SSc patients.

**Methods:**

An integrative gene expression analysis of ten independent microarray datasets of three tissues was conducted to identify differentially expressed genes (DEGs). DEGs were mapped to the search tool for retrieval of interacting genes (STRING) to acquire protein–protein interaction (PPI) networks. Then, functional clusters in PPI networks were determined. Enrichr, a gene list enrichment analysis tool, was utilized for the functional enrichment of clusters.

**Results:**

A total of 12, 2, and 4 functional clusters from 619, 52, and 119 DEGs were determined in the lung, peripheral blood mononuclear cell (PBMC), and skin tissues, respectively. Analysis revealed that the tumor necrosis factor (TNF) signaling pathway was enriched significantly in the three investigated tissues as a common pathway. In addition, clusters associated with inflammation and immunity were common in the three investigated tissues. However, clusters related to the fibrosis process were common in lung and skin tissues.

**Conclusions:**

Analysis indicated that there were common pathological clusters that contributed to the pathogenesis of SSc in different tissues. Moreover, it seems that the common pathways in distinct tissues stem from a diverse set of genes.

## Background

Systemic sclerosis (SSc) is a rare, multisystemic, autoimmune disease that involves the skin and various internal organs, including the lungs, gastrointestinal tract, heart, and kidneys. The exact pathogenesis of SSc remains unknown, but it seems that vascular abnormalities, inflammation, dysregulation of immune system, and extracellular matrix (ECM) deposition can lead to progressive connective tissue fibrosis. Organ failures that arise from fibrosis are the most significant causes of mortality in SSc patients [1, 2].

Although the etiopathogenesis of SSc has not been well identified, accumulated evidence suggests that multiple genes and their interactions with environmental factors play important roles in this context [3, 4]. Traditional researches have been performed in order to demonstrate the involvement of a particular gene or protein in SSc physiopathology [5, 6]. Although these studies generate invaluable data, they provide a small amount of evidence that is insufficient to clarify the complex interactions between multiple genes or proteins simultaneously. Consequently, it is essential to utilize new approaches for realizing the alterations of different genes and pathways in complicated pathological conditions, like SSc [7, 8]. These approaches could have a major role in the holistic understanding of complex disease patterns and developing effective therapies.

Microarrays have been extensively applied for understanding biological mechanisms, discovering new drug targets, and evaluating drug responses [9, 10]. In addition, results obtained from microarray technology might be helpful in generating abundant complex datasets that mostly address the same biological inquiries [11–17]. Integration of relevant gene expression datasets can improve the reliability of the outputs and facilitate the identification of altered molecular pathways and complex disease pathogeneses [8, 18, 19].

Skin involvement is one of the most common clinical manifestations of SSc and is known to be a key marker of disease activity [20]. The lung is frequently involved in SSc, and such condition is known as the major cause of death among SSc patients [21]. PBMC is a valuable resource for investigating the immune responses involved in autoimmune diseases like SSc [22]. The involvement of multiple organs makes it difficult to recognize the SSc pathogenesis. Moreover, it is not yet clearly understood what pathways may affect SSc development in different organs [23]. Consequently, the present study accomplished an integrative analysis of microarray gene expression data of PBMC as well as the lungs and skin of SSc patients to identify the shared and tissue-specific pathways involved in different tissues.

## Methods

### Methods flowchart

**Fig. 1:**
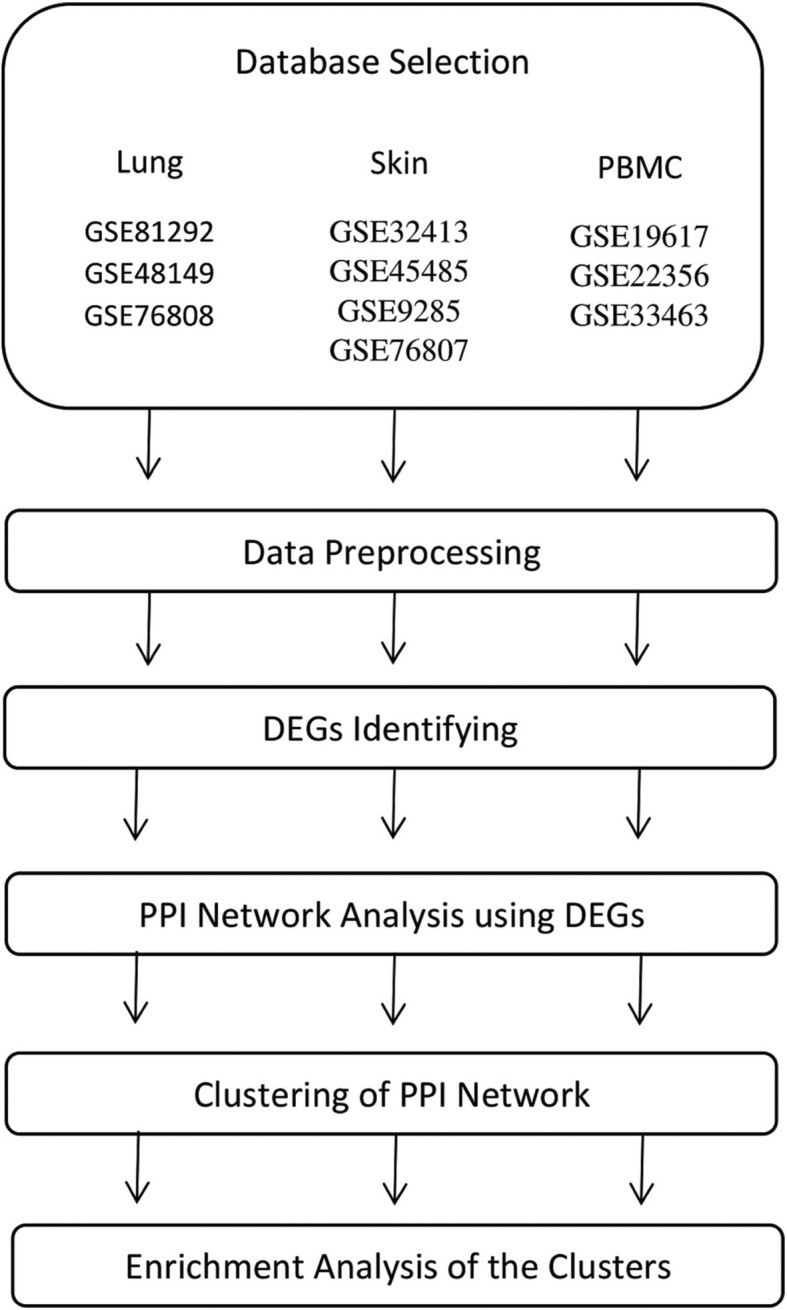
Flowchart of methods

The method procedures and steps are illustrated in Fig. 1.

### Gene expression dataset selection

Gene Expression Omnibus (GEO) (https://www.ncbi.nlm.nih.gov/geo/) was searched for gene expression datasets regarding SSc [24]. Datasets containing case and control samples were selected. In addition, only SSc patients who had received no treatment were included. A total of 10 datasets possessed the selection criteria and were selected for this study. Three datasets for lung tissue (accession number: GSE81292, GSE48149, and GSE76808), three datasets for PBMC (accession number: GSE19617, GSE22356, and GSE33463), and four datasets for skin tissue (accession number: GSE32413, GSE45485, GSE9285, and GSE76807) were selected. The selected datasets comprised 69 (52 cases and 17 controls), 186 (125 cases and 61 controls), and 88 (30 cases and 58 controls) samples for lung, PBMC, and skin, respectively. Table 1 provides detailed information of each dataset and highlights the first author, tissue type, accession number, and references.

**Table 1:** Characteristics of datasets included in this study

First Author	Tissue	GEO Accession	Reference
Christmann R	Lung	GSE81292	[1]
Feghali-Bostwick CA	Lung	GSE48149	–
Christmann R	Lung	GSE76808	[2]
Pendergrass S	PBMC	GSE19617	[3]
Risbano MG	PBMC	GSE22356	[4]
Cheadle C	PBMC	GSE33463	[5]
Pendergrass S	Skin	GSE32413	[6]
Hinchcliff M	Skin	GSE45485	[7]
Milano A	Skin	GSE9285	[8]
Whitfield ML	Skin	GSE76807	–

Abbreviation: *GEO*: Gene Expression Omnibus; *PBMC*: peripheral blood mononuclear cell

### Datasets preprocessing

The data was preprocessed using R statistical programming language. Series matrix files and related annotations for each dataset were obtained from the GEO database. Selected datasets were divided into different groups based on their tissue types. Then, preprocessing steps were carried out in each tissue group independently. The data in each dataset was normalized using a quantile normalization technique function. Raw expression levels were log2 transformed. The mean was applied to replicate expressions of the same participants. To merge datasets in each tissue, probes were converted to the Entrez gene ID. The probes which were assigned to no Entrez ID were removed. The multiple expressions which were assigned to identical Entrez IDs were collapsed to the mean expression using the aggregate function in R. To make gene expression comparable across samples, batches were removed using well-established ComBat function from the SVA R/Bioconductor package [25].

### Identifying differentially expressed genes (DEGs)

DEGs between healthy controls and patients in PBMCs as well as lung and skin tissues were identified using the Limma package [26]. DEGs were considered significant with an adjusted *p*-value< 0.05 based on the false discovery rate (FDR) using the Benjamini-Hochberg (BH) procedure and the logarithm of fold change (logFC) > ± 0.5. A total of 52, 619, and 119 DEGs between the SSc group and healthy controls were identified in PBMC, lung, and skin tissues, respectively. A complete list of DEGs in lung, PBMC, and skin datasets are provided as Additional file 1:Tables S1, Additional file 2:Tables S2, and Additional file 3: Tables S3, respectively. Analysis showed that there was no shared DEGs between all three investigated tissues; however, there were some common DEGs between each pair of tissues (Table 2).

**Table 2:** Shared DEGs between pair tissues

Pair tissues	Shared DEGs
lung - PBMC	*KLF9, CD69, CISH, GP9, JUN, JUNB, JUND, MT2A, NFE2, SPOCK2, RGCC, CCNL1, SIK1*
lung - skin	*COL5A2, COL6A3, COMP, VCAN, DIO2, FBN1, CFI, IGFBP2, IGFBP7, CYR61, JCHAIN, IL6, PTX3, RGS16, SLC14A1, GDF15, SULF1, STEAP1*
PBMC - skin	*IFI27, PLSCR1, CXCR4, IFI44*

Abbreviation: *DEGs*: Differentially Expressed Genes

### Protein-protein interaction (PPI) network analysis

The search tool for retrieval of interacting genes (STRING) (https://string-db.org) database, which integrates both known and predicted PPIs, can be applied to predict functional interactions of proteins [27]. To seek potential interactions between DEGs according to different tissues, the STRING tool was employed. Active interaction sources, including text mining, experiments, databases, and co-expression as well as species limited to “*Homo sapiens*” and an interaction score > 0.4 were applied to construct the PPI networks. Cytoscape software version 3.6.1 was used to visualize the PPI network. To detect highly connected regions of the network, ClusterONE 1.0 software was used based on the following criteria: minimum size = 5, minimum density = 0.05, and edge weights = combined_score. Minimum size is the minimum size of each cluster; minimum density represents the average edge weight within the cluster if missing edges are supposed to have a weight of zero, and edge weights determine the weight of each edge [28]. In the networks, the nodes correspond to the proteins and the edges represent the interactions. STRING was employed to seek potential interactions among DEGs corresponding to different tissues. Active interaction sources, including experimental repositories, computational prediction methods, and public text collections as well as species limited to “*Homo sapiens*” and a combined score > 0.4, were applied.

### Functional and pathway enrichment analysis

Gene Ontology (GO) and Kyoto Encyclopedia of Genes and Genomes (KEGG) pathway enrichment analyses were conducted using Enrichr (http://amp.pharm.mssm.edu/Enrichr/) for clusters obtained from different SSc tissues. Enrichr is a web-based tool that allows the evaluation of annotations with its extensive gene-set libraries [29]. The GO Biological Process 2018 and KEGG 2016 of each tissue were determined. The significant terms and pathways were selected with the threshold of adjusted *p*-value < 0.05. The five most significant (adjusted *p-*value < 0.05) GO biological processes and KEGG pathways in each cluster of lungs, PBMC, and skin datasets are listed in Tables 3, 4, and 5, respectively.

**Table 3:** Most significant GO and KEGG pathways enriched in lung clusters

Go Biological Process	*P*-value	KEGG Pathway	*P*-value
cluster L.1 (Inflammation & immunity) – size/DEGs = 56/54			
- cytokine-mediated signaling pathway	1.65E-17	- Cytokine-cytokine receptor interaction	5.61E-22
- inflammatory response	3.73E-11	- Hematopoietic cell lineage	1.05E-10
- cellular response to cytokine stimulus	1.25E-12	- Chemokine signaling pathway	2.66E-09
- chemokine-mediated signaling pathway	2.78E-10	- JAK-STAT signaling pathway	1.06E-08
- positive regulation of leukocyte migration	6.87E-08	- TNF signaling pathway	3.78E-07
cluster L.2 (Inflammation & immunity) – size/DEGs = 48/47			
- regulation of transcription from RNA polymerase II promoter	1.92E-12	- MAPK signaling pathway	1.48E-09
- positive regulation of transcription from RNA polymerase II promoter	1.04E-10	- TNF signaling pathway	1.04E-05
- positive regulation of transcription, DNA-templated	6.58E-10	- Osteoclast differentiation	2.04E-05
- regulation of cell cycle	1.13E-08	- Inflammatory bowel disease (IBD)	0.000298
- regulation of transcription, DNA-templated	7.42E-06	- Amphetamine addiction	0.000298
cluster L.3 (Cell proliferation & cell death) - size/DEGs = 20/19			
- positive regulation of cyclin-dependent protein serine/threonine kinase activity	0.000225	- p53 signaling pathway	1.44E-07
- positive regulation of cell cycle	0.000225	- Cell cycle	1.4E-06
- mitotic cell cycle phase transition	0.000225	- FoxO signaling pathway	6.39E-05
- G1/S transition of mitotic cell cycle	0.000225	- Progesterone-mediated oocyte maturation	0.000704
- cell cycle G2/M phase transition	0.000284	- Oocyte meiosis	0.001103
cluster L.4 (GPCR signaling) - size/DEGs = 9/9			
- adenylate cyclase-activating G-protein coupled receptor signaling pathway	5.98E-17	- Neuroactive ligand-receptor interaction	7.6E-09
- adenylate cyclase-modulating G-protein coupled receptor signaling pathway	2.53E-15	- Regulation of lipolysis in adipocytes	0.001669
- cAMP-mediated signaling	1.11E-11	- Renin secretion	0.001669
- G-protein coupled receptor signaling pathway, coupled to cyclic nucleotide second messenger	4.58E-08	- Salivary secretion	0.002418
- positive regulation of cAMP metabolic process	5.94E-06	- Vascular smooth muscle contraction	0.003501
cluster L.5 (GPCR signaling) - size/DEGs = 19/19			
- regulation of small GTPase mediated signal transduction	1E-10	- Axon guidance	0.006298
- regulation of intracellular signal transduction	3.27E-07		
- regulation of actin filament-based process	2.44E-05		
- regulation of cell migration	2.44E-05		
- regulation of actin cytoskeleton organization	3.37E-05		
cluster L.6 (ECM organization) - size/DEGs = 13/11			
- extracellular matrix organization	3.55E-11	- Protein digestion and absorption	1.08E-15
- collagen fibril organization	1.56E-07	- AGE-RAGE signaling pathway in diabetic complications	6.03E-05
- protein complex subunit organization	6.15E-07	- Amoebiasis	6.03E-05
- skeletal system development	6.58E-07	- ECM-receptor interaction	6.03E-05
- skin development	6.58E-07	- Platelet activation	8.48E-05
cluster L.7 (Metabolic process) - size/DEGs = 8/7			
- secondary alcohol biosynthetic process	1.83E-08	- Terpenoid backbone biosynthesis	5.16E-07
- cholesterol biosynthetic process	1.83E-08	- Synthesis and degradation of ketone bodies	2.52E-05
- sterol biosynthetic process	1.87E-08	- Steroid biosynthesis	7.07E-05
- cholesterol metabolic process	1.19E-07	- Metabolic pathways	8.42E-05
- acetyl-CoA metabolic process	2.02E-07	- Butanoate metabolism	8.42E-05
cluster L.8 (Regulation of BMPs) - size/DEGs = 20/20			
- regulation of BMP signaling pathway	5.24E-11	- Signaling pathways regulating pluripotency of stem cells	1.69E-09
- BMP signaling pathway	4.87E-08	- Hedgehog signaling pathway	1.8E-08
- cellular response to BMP stimulus	5E-08	- Basal cell carcinoma	1.96E-08
- regulation of ossification	5.47E-08	- Hippo signaling pathway	4.83E-08
- positive regulation of cartilage development	2.94E-07	- TGF-beta signaling pathway	8E-06
cluster L.9 (Metabolic process) - size/DEGs = 11/11			
- keratan sulfate catabolic process	0.00139	- Phospholipase D signaling pathway	0.001041
- keratan sulfate metabolic process	0.002787	- Rap1 signaling pathway	0.001615
- sulfur compound catabolic process	0.002787	- cAMP signaling pathway	0.029672
- keratan sulfate biosynthetic process	0.002787	- Ras signaling pathway	0.029672
- glycosaminoglycan catabolic process	0.005613		
cluster L.10 (GPCR signaling) - size/DEGs = 22/21			
- axon guidance	0.00022	- Axon guidance	1.8E-07
- transmembrane receptor protein tyrosine kinase signaling pathway	0.000396	- Focal adhesion	0.0014420.011273
- axonogenesis	0.000396	- Primary immunodeficiency	0.032572
- cell migration involved in sprouting angiogenesis	0.007527	- B cell receptor signaling pathway	
- ephrin receptor signaling pathway	0.007527		
cluster L.11 (ECM degradation) - size/DEGs = 23/23			
- proteolysis	0.000176	None	
- extracellular matrix disassembly	0.00022		
- regulation of endopeptidase activity	0.005145		
- extracellular matrix organization	0.007401		
- regulation of membrane protein ectodomain proteolysis	0.014697		
cluster L.12 (Inflammation and immunity) - size/DEGs = 12/11			
- neutrophil degranulation	0.004647	- Insulin secretion	0.010314
- neutrophil mediated immunity	0.004647	- Osteoclast differentiation	0.012295
- neutrophil activation involved in immune response	0.004647		
- potassium ion transport	0.014375		
- metal ion transport	0.027277		

Abbreviation: *GPCR*: G-protein coupled receptor; *BPMs*: Bone morphogenetic proteins

**Table 4:** Most significant GO and KEGG pathways enriched in PBMC clusters

Go Biological Process	*P*-value	KEGG Pathway	*P*-value
Cluster P.1 (Inflammation and immunity) - size/DEGs = 7/7			
- type I interferon signaling pathway	1.16E-07	None	
- cellular response to type I interferon	1.16E-07		
- endosomal vesicle fusion	0.000119		
- cytokine-mediated signaling pathway	0.000503		
- negative regulation of viral genome replication	0.001647		
Cluster P.2 (Inflammation and immunity) - size/DEGs = 9/9			
- response to organophosphorus	5.96E-06	- Apoptosis	1.16E-07
- response to purine-containing compound	5.96E-06	- Osteoclast differentiation	6.68E-06
- response to cytokine	1.34E-05	- TNF signaling pathway	0.000265
- cellular response to organic substance	1.34E-05	- Influenza A	0.000798
- response to cAMP	2.61E-05	- Viral carcinogenesis	0.001022

**Table 5:** Most significant GO and KEGG pathways enriched in skin clusters

Go Biological Process	*P*-value	KEGG Pathway	*P*-value
cluster S.1 (ECM organization) - size/DEGs = 11/11			
- extracellular matrix organization	9.32E-10	- Protein digestion and absorption	7.46E-09
- skeletal system development	5.4E-07	- ECM-receptor interaction	5.35E-05
- eye morphogenesis	6.6E-06	- Focal adhesion	0.000526
- collagen fibril organization	1.51E-05	- PI3K-Akt signaling pathway	0.001831
- eye development	3.74E-05		
cluster S.2 (Inflammation and immunity) - size/DEGs = 14/14			
- inflammatory response	3.4E-06	- Legionellosis	3.16E-06
- neutrophil mediated immunity	5.53E-05	- Pertussis	5.59E-06
- cellular response to cytokine stimulus	5.53E-05	- Rheumatoid arthritis	7.79E-06
- response to lipopolysaccharide	0.000268	- TNF signaling pathway	1.31E-05
- positive regulation of apoptotic cell clearance	0.000619	- Phagosome	4.02E-05
cluster S.3 (ECM organization) - size/DEGs = 7/7			
- platelet degranulation	1.5E-08	- Complement and coagulation cascades	0.002555
- regulated exocytosis	1.83E-08		
- extracellular matrix organization	1.08E-07		
- post-translational protein modification	0.003869		
- extracellular matrix disassembly	0.005238		
cluster S.4 (Inflammation and immunity) - size/DEGs = 5/5			
- type I interferon signaling pathway	0.001321	- RIG-I-like receptor signaling pathway	0.01738
- negative regulation of viral genome replication	0.001321		
- negative regulation of viral life cycle	0.001321		
- cellular response to type I interferon	0.001321		
- regulation of viral genome replication	0.001321		

## Results

### Data quality control

**Fig. 2:**
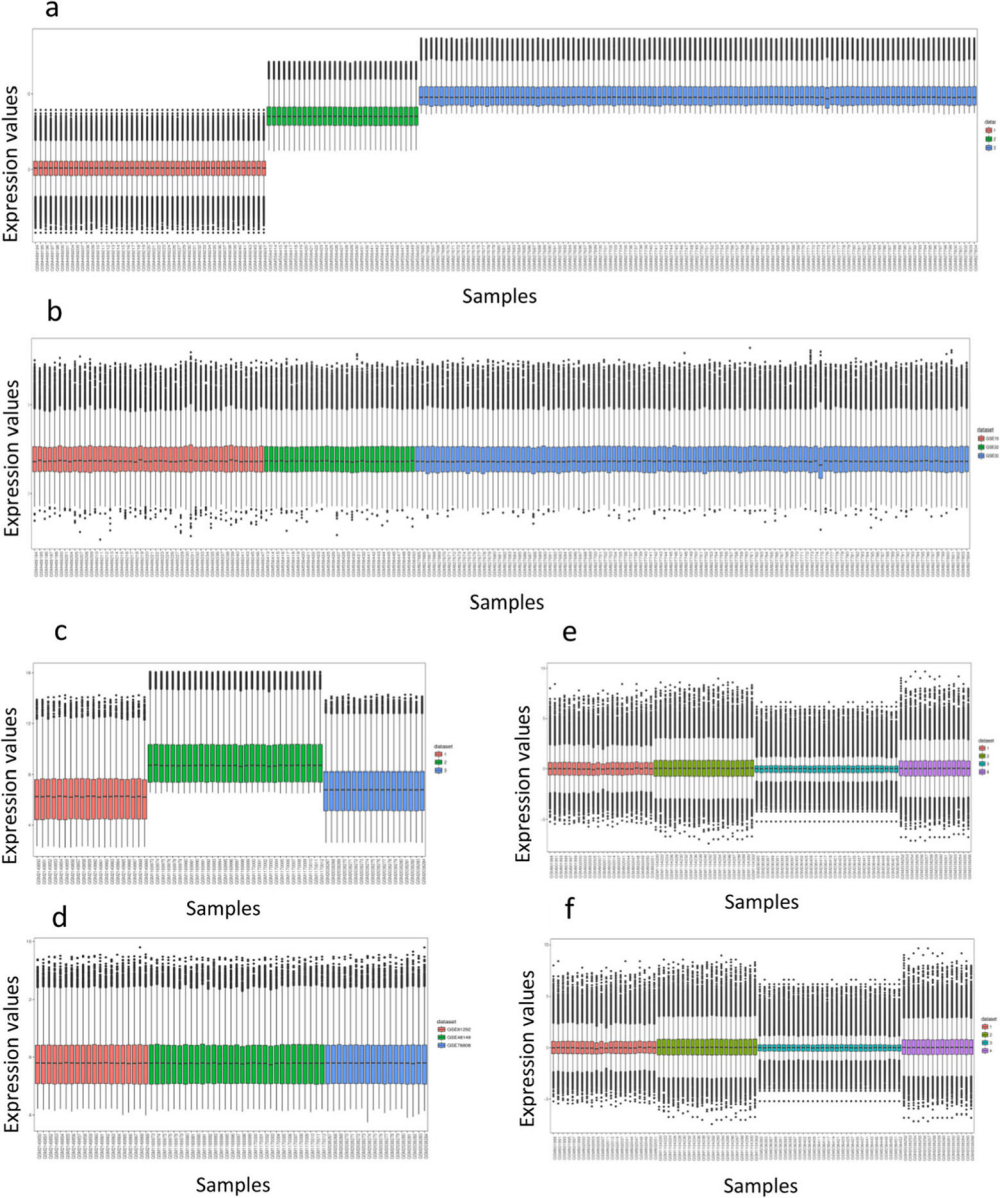
Boxplots of data before and after batch effect correction. **a**) Boxplot of PBMC data before batch effect removal. **b**) Boxplot of PBMC data after batch effect removal. **c**) Boxplot of lung data before batch effect removal. **d**) Boxplot of lung data after batch effect removal. **e**) Boxplot of skin data before batch effect removal. **f**) Boxplot of skin data after batch effect removal. Different datasets were displayed in different colors

To ensure normal distribution of the data, the boxplots for each dataset were displayed. To confirm batch effects removal, the boxplots for each dataset were illustrated after applying the ComBat function. Boxplots before and after batch effect removal are shown in Fig. 2.

### Network analysis of the DEGs

The PPI networks for lung, PBMC, and skin DEGs were constructed and used to identify 2, 12, and 4 clusters of highly interconnected nodes in PBMC, lung and skin tissues, respectively.

### Functional enrichment analysis of clusters

**Fig. 3:**
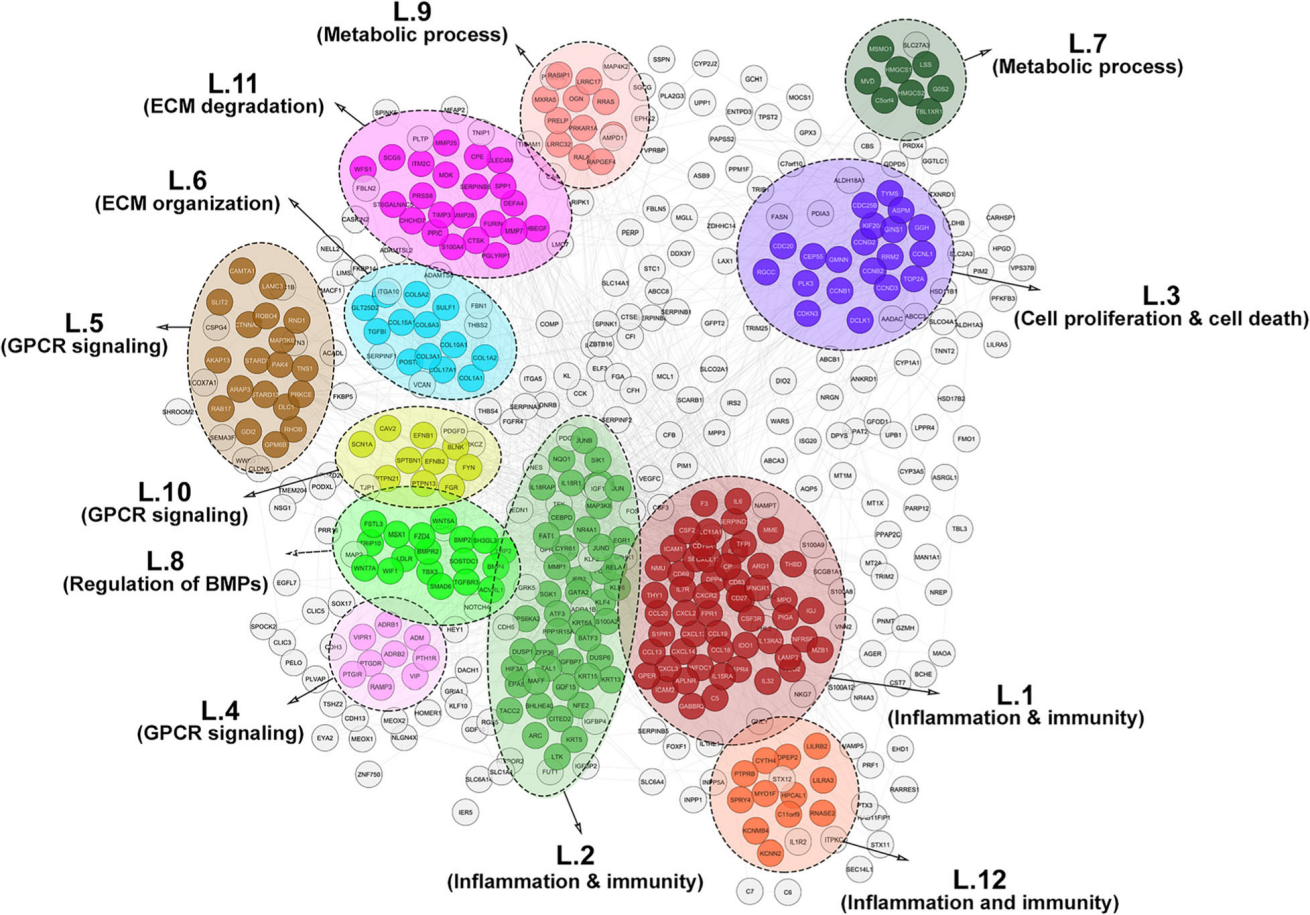
Lung PPI network. Twelve distinct functional clusters were detected in lung tissue. Each cluster is a set of highly-connected nodes and is illustrated in a discrete color

Fig. 3 shows the 12 significant clusters that were found in the lung PPI network analysis using ClusterONE. In the cluster L.1, the most significant biological processes and pathways were associated with immunity and inflammatory responses, including cytokine- and chemokine-mediated signaling pathways, cytokine-cytokine receptor interaction, as well as JAK-STAT and TNF signaling pathways. Cluster L.2 was enriched in immunity pathways containing MAPK and TNF signaling pathways and osteoclast differentiation. Enrichment was also observed in biological processes like transcriptional regulation of the RNA polymerase II gene promoter as well as genes involved in the immune system [30]. Moreover, cluster L.12 was related to the terms and pathways relevant to immunity, including neutrophil degradation, neutrophil-mediated immunity, and osteoclast differentiation. As mentioned, 3 out of 12 clusters of the lung PPI network were correlated with inflammatory and immunity responses.

Several enriched biological processes and pathways in cluster L.3 were involved in cell proliferation and death. For example, mitotic cell cycle phase transition, cell cycle, p53, and FoxO signaling pathways were represented in this cluster. Cluster L.8 was enriched in the regulation of bone morphogenetic proteins (BMPs) and proliferation.

Clusters L.4, L.5, and L.10 all contained biological processes and pathways dependent upon G-protein coupled receptor (GPCR) signaling. GPCRs are a major family of cell surface receptors that are involved in the physiological processes, including regulation of immune systems, cellular motility, and differentiation [31].

The enrichment of ECM organization terms and pathways was observed to be associated with cluster L.6. For example, ECM organization, collagen fibril organization, and protein complex subunit organization terms as well as protein digestion and absorption and ECM-receptor interaction pathways were enriched in cluster L.6. Likewise, cluster L.11 was observed to be more represented in ECM degradation terms, such as proteolysis and ECM disassembly. However, no meaningful KEGG pathways were assigned to this cluster.

As shown in Table 3, clusters L.7 and L.9 were more representative of metabolic terms and pathways, such as the cholesterol metabolic process, synthesis and degradation of ketone bodies, and keratan sulfate metabolic process.

**Fig. 4:**
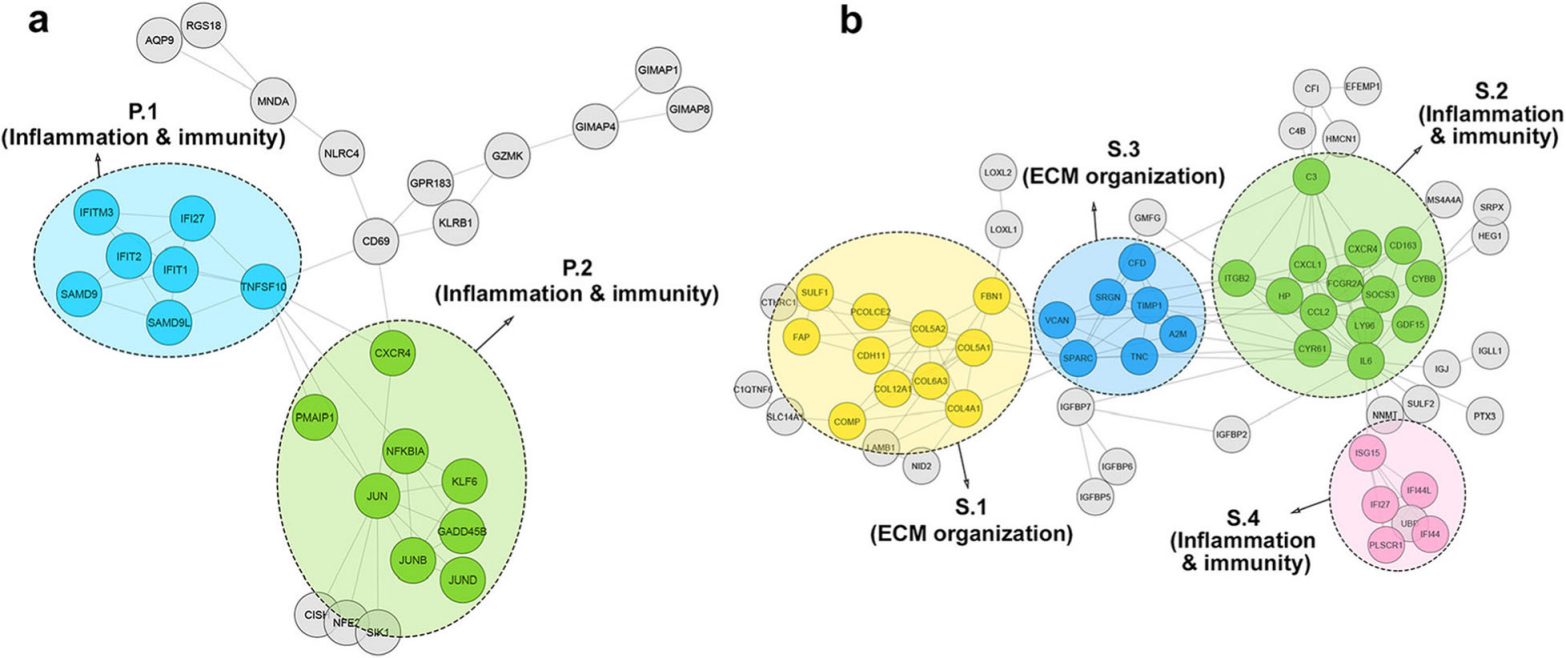
**a**) PBMC PPI network. Two distinct functional clusters were identified in PBMC. **b**) Skin PPI network. Four distinct functional clusters were detected in skin tissue. Each cluster is a set of highly-connected nodes and is illustrated in a discrete color

The PPI network of PBMC was divided into two significant clusters. These two clusters and the biological processes and pathways relevant to the PPI network of PBMC are listed in Table 4. Type 1 interferon and cytokine-mediated signaling pathways were prevalent in cluster P.1. However, there was no significant KEGG pathway related to this cluster. Moreover, the biological processes in cluster P.2 were involved in responses to various substances. Apoptosis, TNF signaling pathway, and osteoclast differentiation were more common KEGG pathways in this cluster. As shown in Fig. 4a, all clusters in the PPI network of PBMC were enriched in terms and pathways related to immunity.

Four significant clusters were found in the skin PPI network. Table 5 shows that cluster S.1 contains terms and pathways related to the extracellular matrix organization. However, clusters S.2 and S.4 were mostly enriched in the immunity biological processes and KEGG pathways. Cluster S.3 represents terms and pathways related to immunity, including complement, coagulation cascade, and platelet degradation, as well as terms related to the ECM organization, comprising ECM organization and ECM disassembly. The PPI network of skin clusters is depicted in Fig. 4b.

### TNF signaling significantly enriched in three investigated tissues

Analysis showed that the TNF signaling pathway was enriched significantly in cluster L.1 and cluster L.2 of lung tissue, cluster P.2 of PBMC, and cluster S.2 of skin tissue. Moreover, it was detected that the TNF signaling arose from the function of different sets of genes in individual tissues. The TNF signaling-related genes in each cluster of the three evaluated tissues are represented in Table 6.

**Table 6:** shared pathway and its related genes in three investigated tissues

Tissue	Cluster Name	Shared Pathway	Genes
Lung	cluster L.1	TNF signaling	*IL6, CSF2, CCL20, CXCL3, SELE, CXCL2, ICAM1*
	cluster L.2	TNF signaling	*JUN, MAP3K8, FOS, JUNB, RELA, IL18R1*
PBMC	cluster P.2	TNF signaling	*NFKBIA, JUN, JUNB*
Skin	cluster S.2	TNF signaling	*SOCS3, IL6, CCL2, CXCL1*

## Discussion

Despite the vast amount of research on SSc, its etiopathogenesis has not yet been fully clarified. Consequently, an effective systemic or targeted therapy does not exist [32]. Genome-wide transcriptional profiling and genome-wide association studies in different tissues from SSc patients have produced valuable information, which can be integrated thoroughly to approach SSc pathophysiology with a comprehensive understanding. Integrative gene expression analysis and the construction of PPI networks can be performed using gene expression data extracted from RNA-seq and microarray. RNA-Seq increases accuracy for low-abundance transcripts [33] and has higher resolution for identifying tissue-specific expressions [34]. However, it is a relatively new method, and there is a small amount of RNA-seq data concerning different tissues of SSc in databases compared to microarray. Therefore, in the present study, microarray data from several SSc tissues was used to investigate whether common pathways influence SSc pathogenesis across affected tissues.

The GO and KEGG pathway analyses of clusters in PBMC as well as lung and skin tissues indicated the clusters contained biological processes and pathways, including extracellular matrix organization, immune response, inflammatory response, cell proliferation, and apoptosis, which may play roles in SSc pathogenesis.

Although no shared DEGs were detected among the three evaluated tissues, TNF signaling pathway was enriched significantly in all three as a common pathway. Therefore, it seems that this common pathway arises from diverse sets of genes in distinct tissues. TNF has a pivotal role in response to infections and in the pathogenesis of different immune-mediated disorders, like rheumatoid arthritis (RA) and spondyloarthritis (SpA) [35, 36]. However, its role in fibrotic disorders like SSc is controversial [37]. Investigations have demonstrated that antagonists of TNF prevent fibrosis in mouse models of silica-induced and bleomycin-induced pulmonary fibrosis [38, 39]. Moreover, progressive lung fibrosis has been indicated in patients with RA after treatment with infliximab (a TNF-α blocker) [40]. Conversely, the anti-fibrotic effects of TNF have been indicated in several in vitro studies [41, 42].

Among the cytokines with increased levels in SSc, transforming growth factor (TGF)-β, interleukin (IL)-6, and IL-4 are considered as main fibrogenic cytokines in this disease. Different immune cell types, such as macrophages, T cells, B cells, and dendritic cells (DCs) have also been implicated in the immunopathogenesis of SSc. The involvement of multiple cytokines, cell types, and organs makes it difficult to clarify the precise pathogenesis of SSc. However, it seems that activation of the immune system and initiation of the autoimmunity trigger the tissue fibrosis [43, 44]. Consistently, analysis in the current study indicated that several biological processes and pathways, such as cytokine-mediated signaling, inflammatory response, and TNF signaling which are involved in immunity and inflammatory processes, were significantly enriched in the three investigated tissues.

Cheadle and colleagues have performed an integrative analysis of microarray data from PAH, pulmonary hypertension (PH), and idiopathic arterial hypertension (IPAH), SSc diseases. Their analysis showed that erythrocyte signature was enriched significantly in PBMCs from PH patients in comparison with SSc and healthy individuals [13]. However, we performed an integrative gene expression analysis only on SSc patients and our analysis revealed that the clusters regarding to the immunity and inflammatory processes and pathways were significantly enriched in PBMCs of the SSc patients.

Although all the biological processes and pathways obtained from the PPI network analysis of PBMC were implicated mostly in clusters that can be labeled as immunity or inflammatory, the lung and skin tissues were enriched in other clusters in addition to immunity and inflammatory clusters. For example, some other lung and skin clusters could be labeled as extracellular matrix (ECM) organization. As expected, the terms and pathways associated with fibrosis containing ECM organization, ECM-receptor interaction, and collagen fibril organization were common in lung and skin tissues. The lung PPI network analysis showed that its terms and pathways not only could be labeled as the inflammation, immunity, and ECM organization, but could also be implicated in cell proliferation and cell death, regulation of bone morphogenetic proteins (BMPs), GPCR signaling, and metabolic processes.

The detection of clusters related to cell proliferation and cell death as well as regulation of BMPs is compatible with previous studies that have indicated enrichment of the cell cycle, proliferation, and p53 signaling in SSc [8]. BMPs are growth factors belonging to the transforming growth factor-β (TGF-β) superfamily which play important roles in cell proliferation, apoptosis, and regeneration after injury. The serum and tissue levels of TGF-β, a major pro-fibrotic cytokine in the pathogenesis of SSc, are elevated in SSc patients [45, 46]. Previous studies have indicated that the balance between TGF-β and BMP signaling is essential and is considerably perturbed in pulmonary fibrosis [47]. A report indicated that increased BMPRII degradation, arising from elevated TGF-β activity, led to impaired BMP signaling in patients with PAH and SSc [48].

The altered expression of G protein-coupled receptors (GPCRs) and their ligands has been associated with multiple immune-mediated disorders, like pulmonary arterial hypertension (PAH) and RA [49, 50]. The pathophysiological mechanisms of SSc, like detrimental vasoconstriction, pro-inflammatory, proliferative, and pro-fibrotic effects, are mediated by angiotensin II (Ang II) and endothelin 1 (ET1) through type I angiotensin II receptor (AT1R) and endothelin I receptor (ETAR), respectively [51]. High levels of autoantibodies against GPCRs like AT1R and ETAR contribute to the pathogenesis of SSc [52].

Recent studies have indicated that there is an association between metabolic pathways and immune-mediated disorders [7, 53, 54]. Metabolic processes regarding SSc have not been investigated profoundly. Blood metabolomics analysis revealed that glycolysis, gluconeogenesis, energetic pathways, degradation of ketone bodies, and pyruvate metabolism are the most important networks in SSc [7]. Analysis in the current study revealed that clusters with several metabolic processes and pathways containing synthesis and degradation of ketone bodies and steroid biosynthesis are associated with SSc lung tissue. However, no metabolic pathway or term was enriched in PBMC and skin tissue.

## Conclusions

Based on the current results, it seems that common pathological pathways contribute to the pathogenesis of SSc in different tissues. However, tissue type may make it susceptible to the initiation of more complicated pathways. Areas for future exploration may include determining the role of TNF signaling pathway in the initiation and progression of SSc, the role of GPCRs in the pathophysiology of SSc, the metabolomics profiling of SSc in different tissues, and the role of the metabolic process in SSc pathogenesis. Ultimately, sampling from diverse patients should be conducted tissue by tissue in different stages of the disease to perform more accurate tissue comparisons and design effective systemic or targeted therapies for SSc in the future.

## Additional Files

**Additional file 1: Table S1.** DEGs in SSc lung

**Additional file 2: Table S2.** DEGs in SSc PBMC

**Additional file 3: Table S3.** DEGs in SSc skin

## Data Availability

The datasets analyzed during the current study are available in the GEO repository under accession numbers: GSE81292, GSE48149, GSE76808, GSE19617, GSE22356, GSE33463, GSE32413, GSE45485, GSE9285, and GSE76807.

## Abbreviations

TGF-β: Transforming growth factor-β
Ang II: Angiotensin II
AT1R: Type I angiotensin II receptor
BMPs: Bone morphogenetic proteins
DEGs: Differentially expressed genes
ECM: Extracellular matrix
ET1: Endothelin 1
ETAR: Endothelin I receptor
GEO: Gene Expression Omnibus
GO: Gene Ontology
GPCR: G-protein coupled receptor
KEGG: Kyoto Encyclopedia of Genes and Genomes
PBMC: Peripheral blood mononuclear cell
PPI: Protein–protein interaction
RA: Rheumatoid arthritis
SpA: Spondyloarthritis
SSc: Systemic sclerosis
STRING: Search tool for retrieval of interacting genes
TNF: Tumor necrosis factor
